# Anterior Crural Neuritis

**Published:** 1913-05-24

**Authors:** 


### NEUROLOGY.

### Anterior Crural Neuritis.

Sciatica is a well-known malady even though its pathology may still be obscure in many instances. Similar symptoms referred to branches of the brachial plexus are now well known; more recently still attention has been drawn by several different authors to a precisely similar affection of the anterior crural nerve, and to this affection the names anterior crural neuritis or anterior cruritis have been applied. Six cases were recorded by Dr. Waterhouse in *St. Bartholomew's Hospital Reports* (Vol. XLVI., p. 65). He points out that the lumbar plexus lies in the substance of the psoas muscle and is formed by the anterior division of the first three and part of the fourth lumbar nerves with a communicating branch from the twelfth dorsal, all of which arise from the upper half of the lumbar enlargement of the spinal cord opposite the eleventh and twelfth dorsal vertebræ. From the plexus thus formed is supplied the skin of the dorsum ilii, the lower part of the abdomen, the groins, the scrotum, the front and sides of the thigh, and the inner aspect of the leg as far as the great toe. The muscles innervated are the flexors of the hip, the extensors of the knee (by the anterior crural nerve), the adductors of the thigh (by the obturator), and the cremasteric (by the genito-crural). Filaments are also given to the sacro-iliac, the hip- and the knee-joints. The nerves which constitute this plexus may be damaged by inflammatory conditions of the spinal meninges, by new growths and tuberculous disease of the spine, by aneurysm of the abdominal aorta or iliac artery, by psoas abscess, or by malignant disease within the abdomen; the obturator nerve, the only branch of the plexus that enters the true pelvis, may in addition be injured by pelvic tumours, by obturator hernia, or by the fœtal head during parturition. Appendicitis, perityphlitis, or on the left side sigmoid diverticulitis, do not appear to receive mention as causes of disordered function of these nerves, though it seems not a little strange that inflammations such as these, attended as they often are by so much plastic and purulent inflammation in the iliac fossa, should not spread to the underlying nerves or lead to their compression during the process of cicatrisation, and perhaps future investigations may show that this is not infrequently the case.

Quite apart, however, from any definite assignable cause, anterior cruritis may be met with in middle-aged or elderly men in whom there may be no causes of pressure upon the lumbar plexus or its branches, the malady being characterised by marked wasting and by loss of power, not amounting to complete paralysis, in the muscles of the front and inner sides of the thigh, together with a complete absence of sensory changes except pain. The patients do not die, so that the precise changes in the affected nerves have not been established clearly, and it is even possible that they are not inflammatory as the name anterior crural neuritis would lead one to suppose; nevertheless, one has no such simple name as sciatica to apply to the affection, and for lack of a better the various cases which represent similar symptoms may be labelled by this name. In not a few cases there is glycosuria, but this is not essential. To judge from Dr. Waterhouse's six cases, together with those recorded by Buzzard, Williamson, and Bruns, it appears that this affection of the lumbar nerves occurs under much the same circumstances and in the same class of patients as the much more common one of sciatica, differing therefrom, however, not only in the area affected, but also to some extent in the greater prominence of the motor as compared to the sensory symptoms.

These symptoms are summarised by Dr. Waterhouse as follows: Subjectively they consist of pain in the district of the sensory supply of the lumbar plexus, together with weakness of the muscles, which flex and adduct the hip and extend the knee. The manner in which a patient indicates the site of pain is very characteristic; he first places the palm of the hand over the front part of the iliac crest, and then sweeps it down over the front of the thigh. Sometimes tender spots are present at a spot about the junction of the upper and middle thirds of the skin on the inner side, and at a point on the inner side of the dorsum of the foot. The effect of the muscular weakness is shown by difficulty in mounting stairs, in rising from the sitting position, and in crossing one leg over the other, whilst in walking there is a sense of insecurity about the knee-joint, which later is apt to flex unexpectedly.

The objective symptoms consist of greater coldness of the surface of the affected thigh, wasting of the muscles of the front and inner side, and diminution or loss of the knee-jerk and cremasteric reflex.

In two cases there was evidence of disease of the corresponding knee-joint, but as in both this was slight, and in one certainly and in the other probably developed after the neuritis, the suggestion that the muscular wasting was the result of the joint disease may be rejected at once. Though these slight articular changes may possibly be evidence of trophic alterations in the joint in consequence of disturbed innervation, the more probable view is that they are the result of internal traumatism of the joint occasioned by the faulty action of the weakened quadriceps. The tendon of this muscle is intimately connected with the capsule of the knee-joint, whilst fibres of the subcrureus are actually inserted into the synovial membrane itself; when the muscle is weakened, the controlling action it exerts on the capsule of the joint during movement is removed, and folds of the synovial membrane are liable to be squeezed and pinched between the component bones. Since the arthritis thus set up may cause further weakening of the quadriceps by reflex atrophy, a vicious circle may arise in these cases just as in cases in which the primary lesion is of the joint.

